# Supplementary material for: Patterns of Intron Gain and Loss in Fungi
Source: PLoS Biol. 2004 Nov 30;2(12):e422. doi: 10.1371/journal.pbio.0020422 (PMC532390; doi:10.1371/journal.pbio.0020422)
Supplement: Table S1 — Also available at http://genes.mit.edu/NielsenEtAl/. (4.3 MB ZIP). [file pbio.0020422.st001.zip › NielsenEtAl/html/1297.html]

AN0327.1.NCU06854.1.MG03173.1.FG06260.1


```
 CLUSTAL W (1.82) Multiple Sequence Alignments - Introns Inserted


Sequence 1: NCU06854.1	481 aa
Sequence 2: MG03173.1	465 aa
Sequence 3: FG06260.1	464 aa
Sequence 4: AN0327.1	452 aa
Alignment Length: 481 aa
Number Identitical Residues: 351 aa
Alignment Score (without introns) 14143


MG03173.1 	---~--MTVSETK~ELRGLSLIAAHSHIRGLGVDADTLEPRAASQGLVGQAKARKAAAVV
NCU06854.1	MAA0QVVTVGESK~DLRGLNLIAAHSHIRGLGVDADTLEPRVASQGLVGQEKARKAAAVV
FG06260.1 	---~------MAA0DLRGLNLIAAHSHIRGLGVDATTLEPRASSQGLVGQEKARKAAAVM
AN0327.1  	---~---------~----MNLIAAHSHIRGLGVDVDSLQPRPASQGLVGQEKARKAAAVI
          	                  :.**************. :*:** :******* ********:

MG03173.1 	LEMIKQGKIAGRAVLIAGPPS~TGKTAIAMGMAQSLGSDVPFTTLASSEIFSLEMSKTEA
NCU06854.1	LEMIKQGKIAGRAVLIAGPPS2TGKTALAMGMAQSLGTDVPFTTLAASEIYSLEMSKTEA
FG06260.1 	LQMIKEGKIAGRAVLIAGPPS2TGKTAIAMGMAQSLGPDVPFTTLASSEIFSLEMSKTEA
AN0327.1  	LQMVKEGKIAGRAVLIAGPPS~TGKTAIAMGMAQSLGPDVPFTMLAASEIFSMEMSKTEA
          	*:*:*:*************** *****:*********.***** **:***:*:*******

MG03173.1 	LTQAFRKSIGVRIKEESEIMEGEVVEIQIDRSVTG~GAKQGKLTIKTTDMEAIYDMGSKM
NCU06854.1	LTQAFRKSIGVRIKEESEIMEGEVVEIQIDRSVTG~HAKQGKLTIKTTDMEAIYDMGSKM
FG06260.1 	LTQAFRKSIGVRIKEESEIMEGEVVEIQIDRSVTG~SAKQGKLTIKTTDMEAVYDMGSKM
AN0327.1  	LTQAFRKSIGVRIKEESEIIEGEVVEIQIDRSVTG0GNKQGKLTIKTTDMETIYDMGTKM
          	*******************:***************   *************::****:**

MG03173.1 	IDAMTKERVMAGDIISIDKSSGKITKLGRSYARSRDYDAMGVDTKFLQCPDGELQKRKEV
NCU06854.1	IDAMTKERVMAGDIISIDKSSGKITKLGRSYARSRDYDAMGVDTKFLQCPDGELQKRKEV
FG06260.1 	IDAMTKERVMAGDIISIDKSSGKITKLGRSYARSRDYDAMGVDTKFLQCPDGELQKRKEV
AN0327.1  	IDSMTKERVMAGDIISIDKSSGKITKLGRSYARSRDYDAMGADVKFVQCPEGELQVRKEI
          	**:**************************************.*.**:***:**** ***:

MG03173.1 	VHTVTLHEIDVINSRTQGFLALFSGDTGEIRSEIRDQINTKVAEWKEEGKADIVPGVLFI
NCU06854.1	VHTVTLHEIDVINSRTQGFLALFSGDTGEIRSEIRDQINTKVAEWKEEGKAEIVPGVLFI
FG06260.1 	VHTVTLHEIDVINSRTQGFLALFSGDTGEIRSEIRDQINTKVGEWKEEGKAEIVPGVLFI
AN0327.1  	VHTVSLHEIDVINSRSQGFLALFSGDTGEIRSEVRDQINVKVAEWKEEGKAEIIPGVLFI
          	****:**********:*****************:*****.**.********:*:******

MG03173.1 	DEVHMLDIECFSYINRALEAELAPIVIMASNRGLSRIRGTDYRSPHGLPLDFLDRTQIIA
NCU06854.1	DEVHMLDIECFSYINRALESDLAPIVIMASNRGHSKIRGTDYKSPHGLPLDFLDRISIIN
FG06260.1 	DEVHMLDIECFSYINRALEDDLAPVVIMASNRGNSRIRGTDYRSPHGLPLDFLDRVAIIN
AN0327.1  	DEVHMLDIECYSYINRALEAELAPIVIMASNRGHSRIRGTTYNSPHGLPLDFLDRVVIVS
          	**********:******** :***:******** *:**** *.************  *: 

MG03173.1 	TQPYSSEDINKILGIRATEEEVELTPDALALLTKIGQEAGLRYASNLITTSQLICAKRRA
NCU06854.1	THSYTPDELRQILTIRAQEEEVDLTPDALALLTKIGAEAGLRYASNLITTSQLICAKRKA
FG06260.1 	THSYTPEEIKQIISIRAQEEEVDVHPDALALLTKIGQEAGLRYASNLITTSQLVSAKRKA
AN0327.1  	TQHYSADEIRQILAIRAQEEEIDLSPDALALLTKIGQESNLRYASNIITTSHLLSQKRKA
          	*: *:.:::.:*: *** ***::: *********** *:.******:****:*:. **:*

MG03173.1 	KQVSVDDVQRSFKLFYDPARSIKFVGESEKRLIGNDGVVDFSVANG----------HGEQ
NCU06854.1	KQVGVEDVQRSFKLFYDPARSVKFVQESEKRLIGSDGVVDFRVNGGATGEPAATAAGGDS
FG06260.1 	KQVEVSDVQRSFQLFYDPARSIKFVAESEKRLIGNTGAVDFSVGAAVSN-------GEEK
AN0327.1  	KEVSVDDVQRSYRLFYDPARSVKFVNQYEQRFIGDQGNVNFTASNG------------DA
          	*:* *.*****::********:*** : *:*:**. * *:* .  .            : 

MG03173.1 	MDTT-
NCU06854.1	MDTSS
FG06260.1 	MDLS-
AN0327.1  	MEIS-
          	*: :
```
